# A lactate metabolism-related signature predicting patient prognosis and immune microenvironment in ovarian cancer

**DOI:** 10.3389/fendo.2024.1372413

**Published:** 2024-03-11

**Authors:** Linhua Zhu, Zhuoqun Lin, Kai Wang, Jiaxin Gu, Xiaojing Chen, Ruizhe Chen, Lingfang Wang, Xiaodong Cheng

**Affiliations:** ^1^ Department of Obstetrics, Women’s Hospital, School of Medicine, Zhejiang University, Hangzhou, China; ^2^ Department of Gynecologic Oncology, Women’s Hospital, School of Medicine, Zhejiang University, Hangzhou, China; ^3^ Department of Obstetrics and Gynecology, Taizhou Hospital of Zhejiang Province Affiliated to Wenzhou Medical University, Linhai, China

**Keywords:** ovarian cancer, metabolism, lactate, immune microenvironment, prognostic signature

## Abstract

**Introduction:**

Ovarian cancer (OV) is a highly lethal gynecological malignancy with a poor prognosis. Lactate metabolism is crucial for tumor cell survival, proliferation, and immune evasion. Our study aims to investigate the role of lactate metabolism-related genes (LMRGs) in OV and their potential as biomarkers for prognosis, immune microenvironment, and immunotherapy response.

**Methods:**

Ovarian samples were collected from the TCGA cohort. And 12 lactate-related pathways were identified from the MsigDB database. Differentially expressed genes within these pathways were designated as LMRGs, which undergo unsupervised clustering to identify distinct clusters based on LMRGs. Subsequently, we assessed survival outcomes, immune cell infiltration levels, Hallmaker pathway activation patterns, and chemotaxis among different subtypes. After conducting additional unsupervised clustering based on differentially expressed genes (DEGs), significant differences in the expression of LMRGs between the two clusters were observed. The differentially expressed genes were subjected to subsequent functional enrichment analysis. Furthermore, we construct a model incorporating LMRGs. Subsequently, the lactate score for each tumor sample was calculated based on this model, facilitating the classification of samples into high and low groups according to their respective lactate scores. Distinct groups examined disparities in survival prognosis, copy number variation (CNV), single nucleotide variation (SNV), and immune infiltration. The lactate score served as a quantitative measure of OV's lactate metabolism pattern and an independent prognostic factor.

**Results:**

This study investigated the potential role of LMRGs in tumor microenvironment diversity and prognosis in OV, suggesting that LMRGs play a crucial role in OV progression and the tumor microenvironment, thus serving as novel indicators for prognosis, immune microenvironment status, and response to immunotherapy.

## Introduction

Ovarian cancer (OV), the most fatal gynecological malignancy and the fifth leading cause of cancer-related mortality among women globally (1), predominantly presents at advanced stages with metastasis beyond the ovary, resulting in a dismal prognosis (2). Despite advancements in surgical techniques and chemotherapy options (3), the 5-year survival rate for ovarian cancer patients remains disappointingly low, hovering around 50% (4). Consequently, there is an urgent imperative to identify novel biomarkers and therapeutic targets specific to OV.

The occurrence and progression of tumors are intricately intertwined with the metabolic reprogramming exhibited by tumor cells, which autonomously regulate the fluxes of their diverse metabolic pathways to meet augmented bioenergetic and biosynthetic demands while mitigating oxidative stress crucial for cancer cell proliferation and survival (5, 6). Lactate is a metabolic by-product generated during glycolysis, the anaerobic conversion of glucose into energy (7). Tumor cells frequently exhibit enhanced glycolytic activity and lactate production, even in the presence of oxygen, which is commonly referred to as the Warburg effect (8). Additionally, The role of lactate as a metabolic feedback regulator and unique signaling molecule has been extensively investigated, shedding light on its involvement in various physiological and pathological processes, including the regulation of energy metabolism, immune response, memory formation, wound healing, and tumor development (7, 9). Moreover, lactate can modulate the tumor microenvironment (TME) by inducing angiogenesis, remodeling the extracellular matrix, and suppressing immune responses (10). Moreover, lactate can hinder the activity and functionality of immune cells such as natural killer cells, cytotoxic T cells, and dendritic cells through pH reduction and interference with signaling pathways (11). Furthermore, lactate can impact the expression and function of immune checkpoints like programmed cell death protein 1 (PD-1), which plays a crucial role in regulating immune response efficacy and immunotherapy effectiveness (12).

The TME encompasses not only tumor cells, signaling molecules, and extracellular matrix but also immune cells, fibroblasts, and bone marrow-derived inflammatory cells closely associated with tumor cells (13). Lactate is a crucial metabolite within the TME (9). As a result of the Warburg effect, tumor cells secrete a substantial amount of lactate into the extracellular microenvironment. Lactate and H+ released by tumor cells in the TME contribute to an acidic TME formation, modulate the metabolism of innate and adaptive immune cells, and hinder the activation and proliferation of CD8+ T cells, natural killer (NK) cells, as well as dendritic cells. Lactate can also affect the expression and function of immune checkpoints, such as PD-1, which are involved in the regulation of immune response and the efficacy of immunotherapy (12).

Given the pivotal role of lactate metabolism in ovarian cancer and its potential as a therapeutic target, our study aimed to investigate the expression and prognostic significance of lactate metabolism-related genes (LMRGs) in ovarian cancer. Additionally, we analyzed the correlation between LMRGs and immune microenvironment characteristics, as well as their impact on the effectiveness of immunotherapy. Our study provides novel insights into molecular and clinical characteristics of ovarian cancer subtypes based on lactate metabolism while suggesting potential biomarkers and therapeutic strategies.

## Materials and methods

### Acquisition of data and preprocessing

The TCGA OV RNAseq dataset, along with the phenotype and survival data of patients, was utilized in this study (14) (https://portal.gdc.cancer.gov/). Additionally, the Gene Expression Omnibus (GEO) database (https://www.ncbi.nlm.nih.gov/geo/query/acc.cgi?acc=GSE140082) GSE140082 queue provided OV RNAseq gene expression and patient survival information. Furthermore, the gencode annotation was obtained from the European Bioinformatics Institute (EBI) website (https://www.ebi.ac.uk/). 63 immune checkpoint genes were sourced from the literature (15). 58 chemokines and 12 lactate metabolism-related pathways were acquired from the Molecular Signatures Database (MsigDB) database (http://www.gsea-msigdb.org/gsea/msigdb). **Table 1** summarizes detailed clinical data of OV patients in the TCGA database, while **Table 2** presents comprehensive clinical information on OV patients in the GSE140082 database.

**Table 1:** Clinical characteristics of OV patients in TCGA database.

Characteristics		Number
lymphatic_invasion	YES	180
	NO	109
	N/A	442
age	≥60	344
	<60	387
stage	Stage i	30
	Stage ii	44
	Stage iii	550
	Stage iv	102
	N/A	5
neoplasm_histologic_grade	G1	10
	G2	101
	G3	601
	G4	1
	GB	2
	GX	10
	N/A	6
TCGA.Subtype	OVCA.Differentiated	178
	OVCA.Immunoreactive	128
	OVCA.Mesenchymal	126
	OVCA.Proliferative	165
	N/A	134

N/A, Not Applicable.

**Table 2:** Clinical characteristics of OV patients in GSE140082 database.

Characteristics		Number
age	≥60	181
	<60	199
figo_stage	I	20
	II	31
	III	266
	IV	63
newgrade	high.grade	281
	low.grade	74
	N/A	25

N/A, Not Applicable.

### Identification of the LMRGs in OV

The MsigDB database (16) provided 12 lactate metabolism-related pathways, and the GSVA (17) and GSEABase packages(c5.all.v7.4.symbols.gmt) were used to calculate the ontology enrichment scores of each sample in the TCGA-OV database on these 12 pathways. T-test was employed to determine the enrichment differences between tumor and normal samples, resulting in the identification of 325 genes with significant differences. Among them, a total of 276 protein-coding genes were defined as LMRGs.

### Unsupervised clustering of LMRGs

Based on the LMRGs, an unsupervised clustering method was employed to categorize OV patients in TCGA into geneCluster A and geneCluster B. The “ConsensusClusterPlus” package in R was utilized for performing a consensus clustering algorithm to determine the number of clusters and their stability (18). The optimal number of clusters was selected based on the consensus matrix, cumulative distribution function (CDF), and relative change in the area under the CDF curve.

### Consensus clustering of DEGs

To gain a deeper understanding of the distinct pathways of the clusters, we performed an analysis using the “limma” package in R to compare the expression levels of differentially expressed genes (DEGs) within 2 lactate clusters (19). The significance threshold was set at | logFC | > 0.8 and *p < 0.05*. Subsequently, a Consensus Clustering method (clusterAlg= “hc”, distance= “spearman”) based on DEGs was employed to classify TCGA OV patients into various lactate gene clusters.

### Functional enrichment analysis

The WebGestalt database (http://www.webgestalt.org/) was utilized for Gene Ontology (GO) annotation and Kyoto Encyclopedia of Genes and Genomes (KEGG) (20)pathway enrichment analysis (21). The clusterProfiler (22) and org.Hs.eg.db packages were employed to conduct GO and KEGG pathway enrichment analysis on these differential genes.

### Construction of lactatescore and evaluation of effectiveness

Univariate Cox regression analysis was conducted to identify differentially expressed genes between the two lactate clusters. This analysis revealed 9 genes significantly associated with prognosis (*p<0.01*). Subsequently, principal component analysis (PCA) was performed using the expression levels of these nine genes. The sum of the first two principal components for each sample (lactate_score=∑(PC1i+PC2i)) was utilized as the lactate score. The survminer package was utilized for the classification of patients into high and low-risk groups based on lactate score. Both survminer and survival packages were employed to generate survival curves depicting High lactate score and Low lactate score in TCGA-OV and GSE140082 datasets, respectively. Additionally, the timeROC package was utilized to plot the ROC curve of the TCGA-OV dataset over 1-3 years.

### Comprehensive analysis

The RCircos package was utilized to plot the chromosomal locations of LMRGs. PCA analysis on normal and tumor samples in the TCGA-OV dataset, based on LMRGs, was performed using the pca3d package. The Cytoscape package was employed to visualize the association relationships among LMRGs, while autocorrelation assessment of LMRGs was conducted using the Hmisc package. Hallmark pathway enrichment scores for each sample in the TCGA-OV dataset were calculated using the GSVA and GSEABase packages (h.all.v7.4.symbols.gmt), along with gene signature-based (23) enrichment scores for each sample. Enrichment differences between lactate clusters were computed and compared using the ggplot2 and ggpubr packages.

### Gene mutation analysis

The mutations of genes regarding copy number variation (CNV) and single-nucleotide variation (SNV) in OV patients were explored by the online database Gene Set Cancer Analysis (GISTIC)(https://cloud.genepattern.org/gp/pages/index.jsf?lsid=urn:lsid:broad.mit.edu:cancer.software.genepattern.module.analysis:00125:6.15.28).

### Measurement of immunocyte infiltration

The CIBERSORT algorithm (24) was used to measure the infiltration score of 22 common immunocytes. The relationships between risk score and immunocyte infiltration were measured by the Spearman coefficients using the “Hmisc” R package. The R ggplot 和ggpubr package was used to measure the infiltration score of 21 common immunocytes.

### Statical analysis

The R software (version 3.6.3) was utilized for data analysis. To assess the impact of identified LMRGs on patients’ prognosis, Kaplan-Meier plotter analysis was employed. Univariate and multivariate Cox regression analyses were conducted to evaluate the influence of various factors on patients’ prognosis. Statistical significance levels were defined as follows: ns denoting not significant; *P < 0.05; **P < 0.01; ***P < 0.001; ****P < 0.0001, with ns indicating no statistical significance.

## Results

### Identification of LMRGs

**Figure 1:**
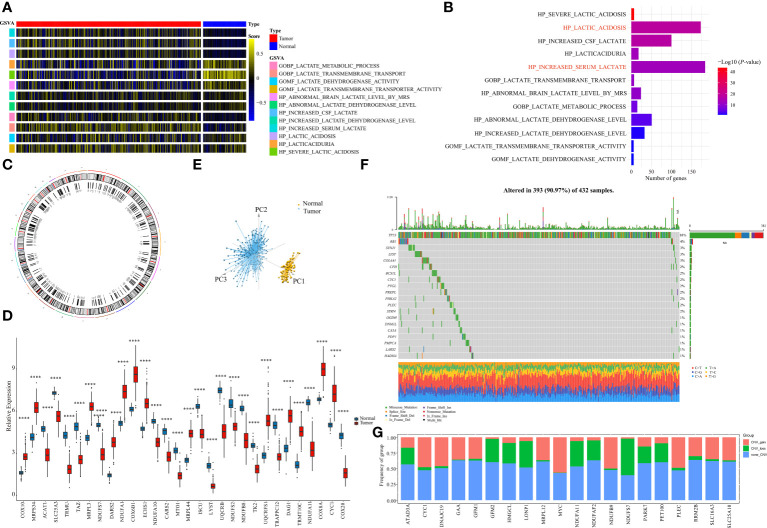
Identification of LMRGs. **(A)** Ontology enrichment score heat map of 12 lactate metabolism-related pathways in the TCGA-OV. **(B)** 10 lactate metabolism-related pathways exhibiting statistically significant differences (*p<0.05*). **(C)** Locations of the LMRGs on 23 chromosomes. **(D)** Boxplot of the expressions of the top 20 LMRGs in the TCGA-OV cohort. **(E)** Principal components analysis (PCA) of the normal samples and the tumor samples based on LMRGs. **(F)** Mutation frequency of the top 20 of 276 differentially expressed LMRGs in 432 patients with OV. **(G)** Copy number variations (CNVs) of the top 20 of 276 differentially expressed LMRGs. ****P < 0.0001.

The MsigDB database was used to explore the lactate-related pathways, and a total of 12 lactate-related pathways were identified as follows: GOBP LACTATE METABOLIC PROCESS, GOBP LACTATE TRANSMEMBRANE TRANSPORT, GOMF LACTATE DEHYDROGENASE ACTIVITY, GOMF LACTATE TRANSMEMBRANE TRANSPORTER ACTIVITY HP ABSOLUTE BRAIN LACTATE LEVEL BY MRS, HP ABSOLUTE LACTATE DEHYDROGENASE LEVEL, HP CREATE CSF LACTATE, HP INCREASED LACTATE DEHYDROGENASE LEVEL, HP INCREASED SERUM LACTATE, HP LACTIC ACIDOSIS, HP LACTICACIDURIA, HP SEVERE LACTIC ACIDOSIS (**Figure 1A**). Their enrichment differences in tumor and normal samples from the OV-TCGA cohort were demonstrated in **Figure 1B**. Among them, the pathway of HP_SEVERE_LACTIC_ACDOSIS obtained the most significant differences between tumor and normal samples.

**Figure 2:**
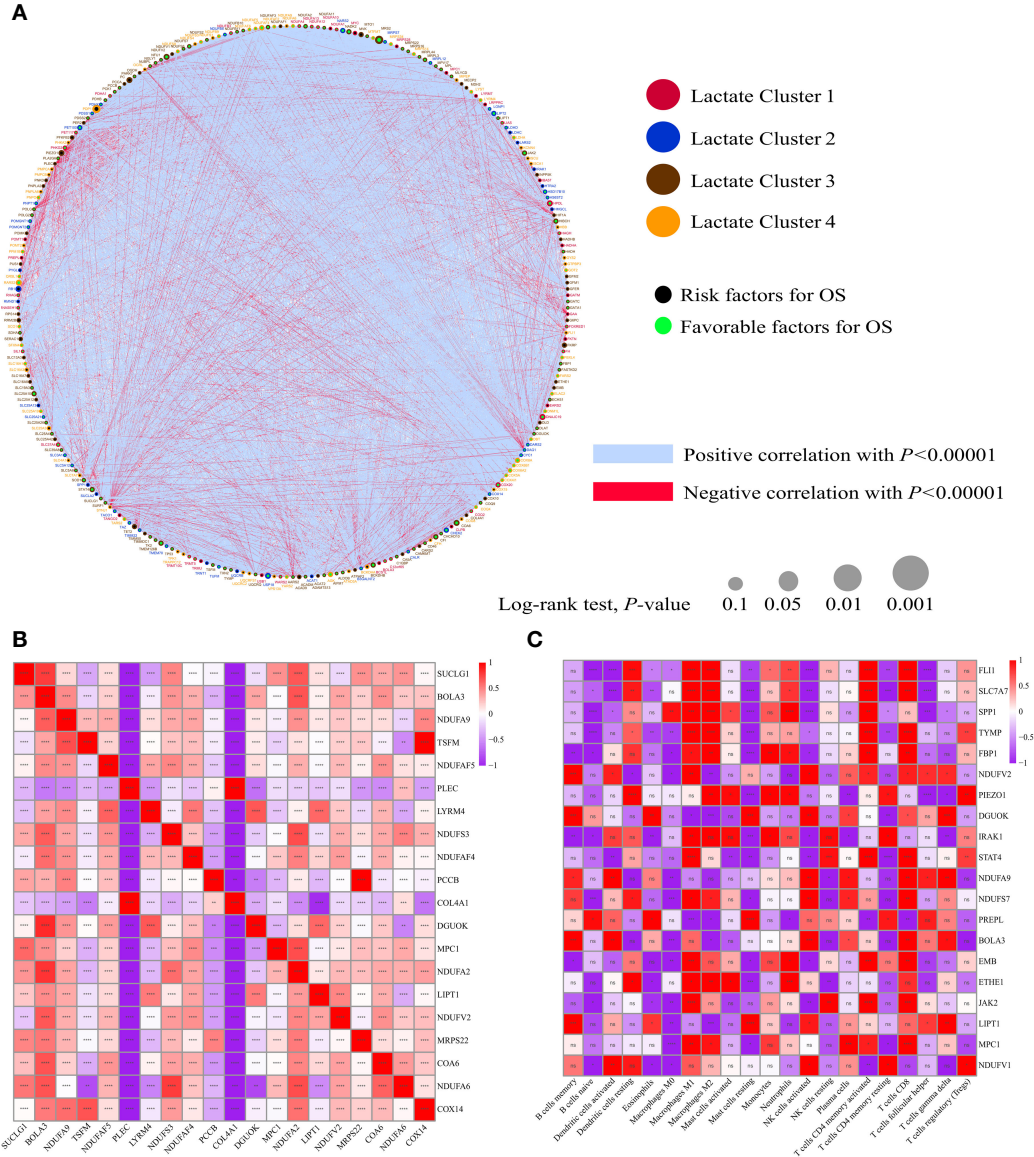
Biological characterizations of LMRGs. **(A)** Correlation and prognostic value of LMRGs in OV. The line connecting the LMRGs represents their correlation, with the line thickness indicating the strength of the correlation between LMRGs. Blue and red represent negative and positive correlations, respectively. **(B)** The autocorrelation of LMRGs is shown in a figure with the top 20 genes represented in both rows and columns. Red indicates a positive correlation, blue indicates a negative correlation. **(C)** The correlation between LMRGs and immune infiltrating cells, with red indicating positive correlation and blue indicating negative correlation. *P < 0.05; **P < 0.01; ***P < 0.001; ****P < 0.0001. ns, no significance.

A total of 325 genes involved in these lactate-related pathways were collected, out of which 276 were protein-coding genes. Their locations on the 24 human chromosomes are displayed in **Figure 1C**. The top 20 genes with significant differences are displayed in **Figure 1D**. Principal components analysis (PCA) revealed that the normal samples could be separated from the tumor samples based on these genes (**Figure 1E**). We then further measured their gene mutation characteristics in OV-TCGA samples (**Figure 1F**). Among them, TP53 was the most frequently mutated gene, with a mutation frequency of approximately 88%. Their CNV mutation characteristics were also measured (**Figure 1G**). The relationships between the regulation of genes related to lactate metabolism and their effects on patient prognosis are displayed in **Figure 2A**. The correlations between the top 20 genes related to lactate metabolism were displayed in **Figure 2B**, and their effects on immune cell infiltration were also measured (**Figure 2C**).

### Unsupervised clustering and subtype analysis based on LMRGs

**Figure 3:**
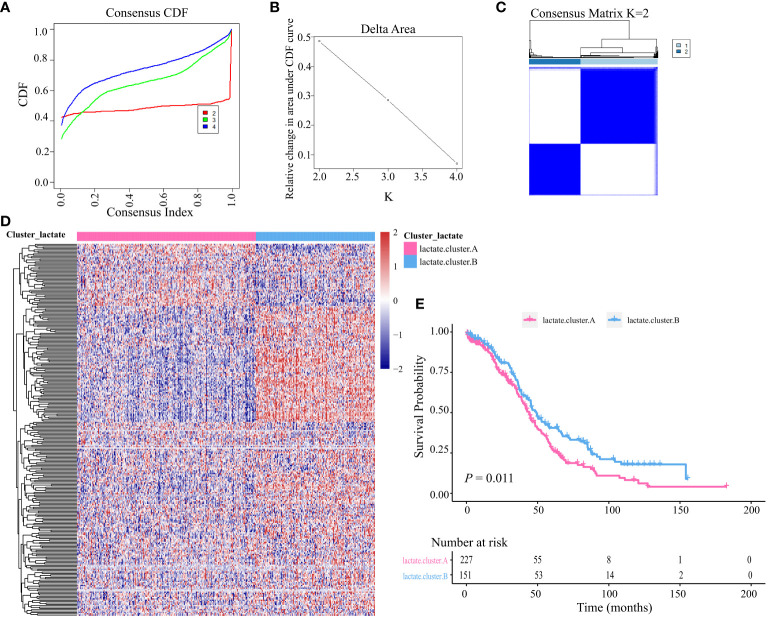
Unsupervised clustering based on LMRGs. **(A)** Cumulative distribution function (CDF) of consensus clustering at k = 2–4. **(B)** relative changes in area under the CDF curve. **(C)** Unsupervised clustering of the 204 differentially expressed LMRGs and optimal consensus matrices for k =2. **(D)** Differences in expression levels of LMRGs between lactate clusters A and B. **(E)** Kaplan-Meier analysis estimating the overall survival between lactate clusters A and B.

Based on the identified LMRGs, we divided all OV-TCGA patients into two subtypes using unsupervised clustering (**Figures 3A–C**). The heatmap showed the expression of clusters A and B (**Figure 3D**), which indicated their vast differences in gene expression profile. Furthermore, Kaplain-Meier survival curve analysis revealed that patients of cluster A significantly obtained unfavorable prognosis compared to those of cluster B (*P = 0.011*, **Figure 3E**).

**Figure 4:**
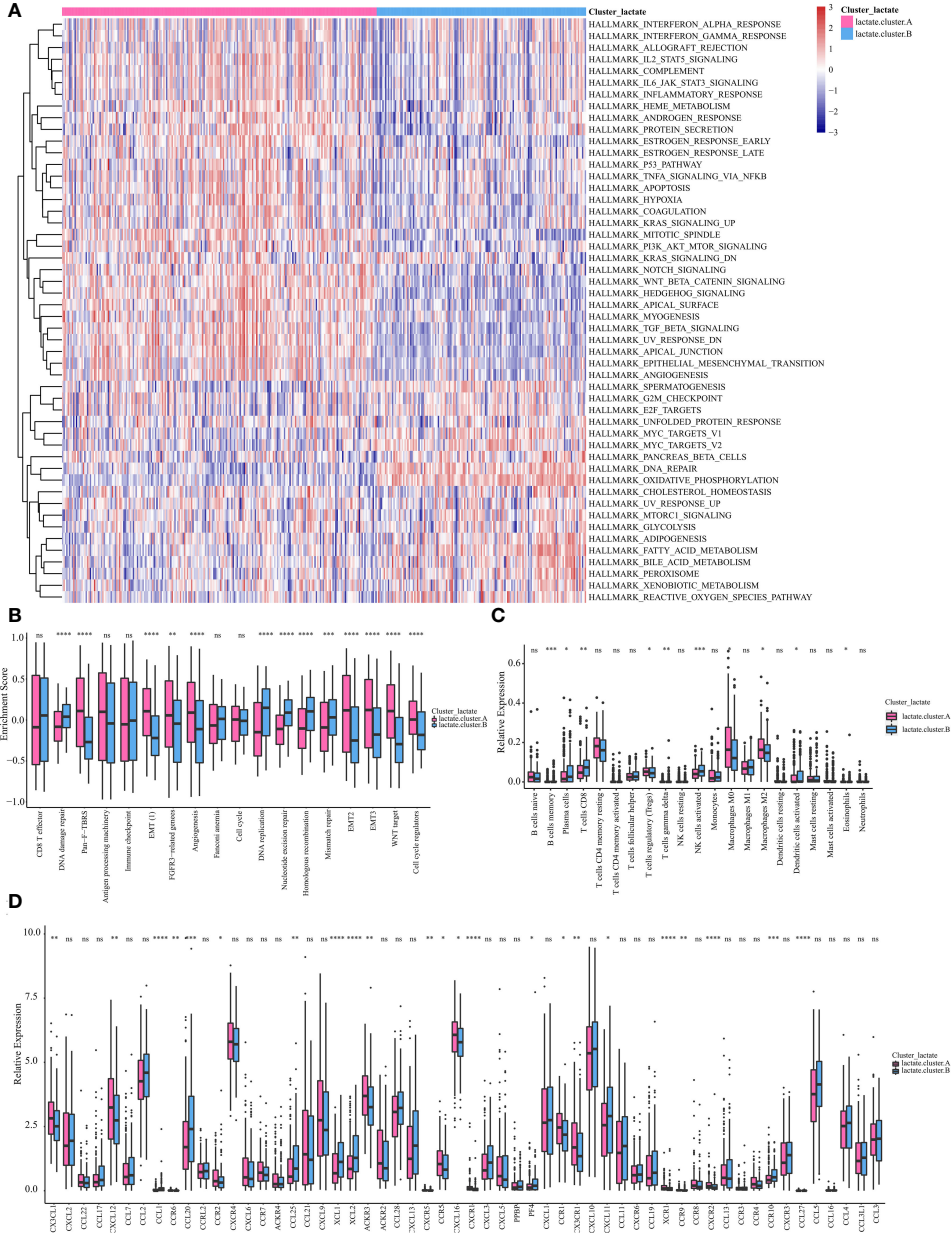
Subtype analysis based on LMRGs. **(A)** GSVA and GSEA analysis focused on the differential enrichment of Hallmaker pathways between lactate clusters A and B. **(B)** Boxplot of the differential enrichment of 13 classic gene signatures between lactate clusters A and B. **(C)** Boxplot of the relative expression of 21 infiltrating immune cells between lactate clusters A and B. **(D)** Boxplot of the relative expression of 57 chemokines between lactate clusters A and B. *P < 0.05; **P < 0.01; ***P < 0.001; ****P < 0.0001. ns, no significance.

We then performed a GSVA score of 50 cancer hallmarks to reveal the difference between the two clusters. As shown in **Figure 4A**, there were obvious differences in certain cancer hallmarks between these two groups. For example, the immune-related pathways (e.g., interferon-alpha response, IL2 stat5 signaling, complement, inflammatory response) were active in cluster A, while the metabolism (e.g., glycolysis, bile acid metabolism, fatty acid metabolism) and cell-cycle related (e.g., G2M checkpoint, E2F targets) pathways were active in cluster B. Comparison of these two subtypes in 13 classic signaling pathways also revealed their vast differences in function (**Figure 4B**). A comparison of these two subtypes in immune infiltration revealed that the infiltering abundance of plasma cells, CD8+ T cells, Tregs, activated NK cells, and DC cells in cluster B were significantly higher than that in cluster A (**Figure 4C**). Correspondingly, the expression of certain chemokines was also differentially expressed in these two subtypes (**Figure 4D**).

### Identification of geneClusters in OV

**Figure 5:**
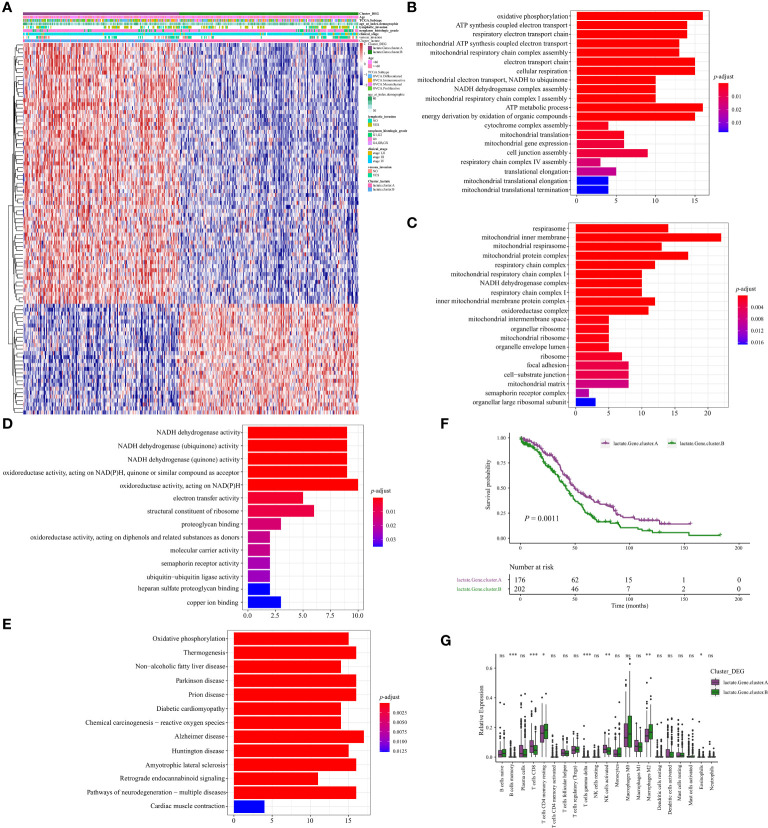
Identification of geneClusters in OV. **(A)** Heatmap illustrating the differences in clinicopathologic features and expression levels of lactate-Phenotype genes between the lactate.Gene.cluster A and B. **(B)** The differential enrichment of GO BP between the lactate.Gene.cluster A and B. **(C)** The differential enrichment of GO CC between the lactate.Gene.cluster A and B. **(D)** The differential enrichment of MF between the lactate.Gene.cluster A and B. **(E)** The differential enrichment of KEGG pathways between the lactate.Gene.cluster A and B. **(F)** Kaplan-Meier analysis estimating the overall survival between lactate.Gene.cluster A and B. **(G)** Boxplot of the relative expression of 21 infiltrating immune cells between the lactate.Gene.cluster A and B. *P < 0.05; **P < 0.01; ***P < 0.001. ns, no significance.

We then identified 94 DEGs (Differentially expressed genes) between clusters A and B and constructed two geneClusters (geneCluster A and geneCluster B) for OV based on unsupervised clustering (**Figure 5A**). Further analysis identified 80 DEGs between geneCluster A and geneCluster B. Functional enrichment these 80 DEGs were mainly involved in the pathways of metabolism (oxidative phosphorylation, ATP synthesis coupled electron transport, ATP metabolic process, NADH dehydrogenase complex) regarding (**Figures 5B–E**). Kaplain-Meier survival curve analysis revealed that patients of geneCluster A significantly obtained better prognosis compared to that of geneCluster B (*P = 0.0011*, **Figure 5F**). Infiltrating abundance analysis of immune cells revealed that there were vast differences between geneCluster A and geneCluster B (**Figure 5G**). For example, CD8+T cells, and activated NK cells were more abundant in geneCluster A. While M2 macrophages were more abundant in geneCluste B. Based on the aforementioned findings, we propose that lactate plays a pivotal role in the characterization of immune infiltration.

### Lactate score was an independent prognostic risk factor in OV

**Figure 6:**
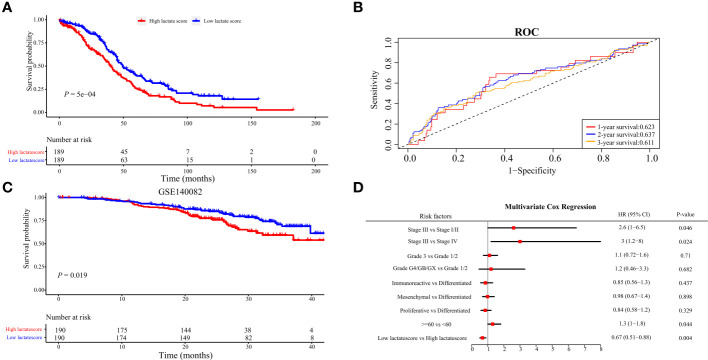
Construction of lactatescore. **(A)** Kaplan-Meier analysis in high versus low lactatescore patients. **(B)** ROC curves to predict sensitivity and specificity of the lactate score in predicting 1-, 2-, and 3-month survival. **(C)** Kaplan-Meier analysis of both groups in the GSE140082. **(D)** lactatescore, TCGA molecular classification, age, stage, and grade were analyzed by multivariate Cox regression analysis.

**Figure 7:**
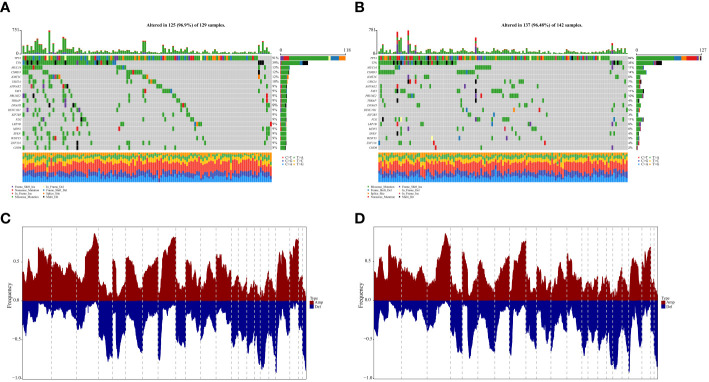
Genomic states of difference between high- and low-lactatescore. **(A, B)** Mutated genes in the TCGA-OV samples of low and high lactatescore subgroups, respectively **(C, D)** Amplify and delete in copy numbers in groups with low and high lactatescore subgroups.

Based on the identified 94 DEGs between clusters A and B, nine pivotal genes: PSMA2, LRP1, TXNDC17, TMEM258, BLOC1S1, HINT2, SOGA1, COA6, and DPM3, which have a significant impact on patient prognosis, were carefully selected to calculate the patient lactate score. As shown in **Figure 6A**, patients with high lactate scores significantly obtained unfavorable prognosis in the OV-TCGA cohort (*P = 5e-04*) compared to those with low lactate score, which were further validated in the GSE140082 cohort (*P = 0.019*, **Figure 6C**). ROC curve displayed the lactate score had good diagnostic efficiency in predicant patients with 1/2/3-year survivals (**Figure 6B**). Multivariate COX regression analysis revealed that low lactate score was an independent prognostic factor for OV patients (HR = 0.67, 95%CI = 0.51-0.88, *P = 0.004*, **Figure 6D**). Further analysis of gene mutations revealed that patients in the high and low lactate score groups exhibited similar patterns of mutations. The TP53, TTN, and MUC16 genes were found to be the most frequently mutated genes in these groups (**Figures 7A, B**). Their CNV patterns were also measured (**Figures 7C, D**).

## Discussion

OV is a prevalent gynecological malignancy worldwide. From a cellular perspective, OV can be classified into epithelial and non-epithelial tumors, with epithelial ovarian cancer accounting for approximately 90% of cases (25, 26). The epithelial subtype further encompasses four distinct histological variants: serous, endometrioid, mucinous, and clear cell types (27, 28). Currently, available therapeutic approaches for OV are limited to radical surgery and chemotherapy regimens that primarily prolong disease-free intervals without significantly impacting overall patient survival rates (29, 30). Therefore, there is an urgent need to establish effective stratification mechanisms along with reliable prognostic indicators to facilitate informed selection among various treatment modalities.

In the tumor microenvironment, cancer cells undergo metabolic adaptations to sustain tumorigenicity and withstand the cytotoxic effects of chemotherapy and immune cell-mediated attacks (31, 32). Alterations in metabolic pathways not only facilitate the survival and proliferation of ovarian cancer cells but also confer abilities for metastasis, chemoresistance acquisition, maintenance of cancer stemness traits, and evasion from immunological surveillance against tumors (33, 34). Since the discovery of lactate in 1780, it has often been mistakenly perceived as a metabolic waste product under hypoxic conditions, leading to numerous detrimental effects and its association with hypoxia (35). Currently, our understanding of lactate has expanded beyond its role as a byproduct of glycolysis to encompass that Lactate is now acknowledged as a pivotal carbon source for cellular metabolism and a crucial signaling molecule in physiological, chronically inflamed, and neoplastic tissues (36). Studies have substantiated that lactate functions as a signaling molecule, playing an indispensable role in coordinating signal transmission among diverse cells, organs, and tissues (37). Lactate is an essential substrate for various physiological cellular functions and exerts regulatory influence on different aspects of energy metabolism and signal transduction (7, 38). Therefore, LMRGs are potential biomarkers and therapeutic targets for various types of cancer (39–44). In hepatocellular carcinoma (HCC), a novel prognostic signature composed of 6 key lactate metabolism-related genes (FKTN, PDSS1, PET117, PUS1, RARS1, and RNASEH1) was developed to predict the survival and tumor microenvironment of HCC patients (44). In endometrial cancer (EC), a risk signature based on 18 lactate metabolism-related genes was constructed to predict the clinical outcome and molecular characteristics of EC patients (40). The role of lactate metabolism-related genes in kidney renal clear cell carcinoma (KIRC) is explored by analyzing the expression patterns of 17 lactate metabolism-related genes in KIRC patient datasets (43). Three lactate metabolism-related genes (FBP1, HADH, and TYMP) were selected from the panel of 17 genes as they showed significant associations with prognosis in KIRC patients (43). These three genes were used to construct a lactate-related prognostic signature that could predict overall survival, tumor microenvironment status, and immune response in KIRC patients (43). In this study, we observed significant activation of metabolism-related pathways (e.g., glycolysis, bile acid metabolism, fatty acid metabolism) in cluster B, which exhibited a more favorable prognosis. Functional enrichment analysis of differentially expressed genes between gene clusters A and B revealed their predominant involvement in metabolic pathways (oxidative phosphorylation, ATP synthesis coupled electron transport, ATP metabolic process, NADH dehydrogenase complex). Kaplan-Meier survival curve analysis demonstrated a significant difference in the prognoses of gene clusters A and B. These findings suggest that metabolism-related pathways play a crucial role in tumor development.

The molecular subgroups of high-grade serous ovarian carcinoma (HGSOC) have been distinguished by research, differentiating between low- and high-OXPHOS phenotypes. High-OXPHOS HGSOCs rely on oxidative phosphorylation as their primary metabolic pathway, while low-OXPHOS HGSOCs exhibit a preference for glycolysis. Additionally, the high-OXPHOS cells demonstrate increased chemosensitivity and a more favorable prognosis (45). In fast-growing cancer cells, there is often a shift from less efficient ATP production through aerobic glycolysis to OXPHOS (46). However, the single-cell multi-omics sequencing analysis of HGSOC revealed a simultaneous enhancement in the activities of both the oxidative phosphorylation pathway and glycolysis pathway during tumor development. Interestingly, mitochondrial oxygen consumption was not completely shut down in tumor cells but rather maintained as a backup system for OXPHOS activity (5). Further analysis indicated that an increase in chromosome 8 copy number during HGSOC development resulted in elevated MYC gene dosage, thereby enhancing the activities of both oxidative phosphorylation and glycolysis pathways (47).

Tumor cells modulate lipid absorption and stimulate lipogenesis to augment intracellular lipid abundance (48), thereby meeting the heightened energy demands for cell proliferation (49). The high prevalence of omental metastasis in ovarian cancer suggests that a lipid-rich environment may facilitate ovarian growth. Further investigations have revealed the upregulation of fatty acid-binding protein 4 (FABP4) in ovarian cancer with momentum metastasis, and its depletion significantly impedes the dissemination of ovarian cancer (49) thus confirming the crucial role of lipid uptake in the progression and advancement of this disease (50).

The intricate TME exerts a profound impact on both the progression of tumors and their response to therapeutic interventions. Currently, numerous studies are dedicated to elucidating the regulatory effects of metabolites and metabolic processes on immune function (51). Among these, lactate emerges as the most significantly elevated metabolic substance in tumors (52). The generation of lactate is generally perceived as a mechanism employed by tumor cells to evade immune surveillance (53, 54). In this study, a comparison of immune infiltration between clusters A and B demonstrated that cluster B exhibited significantly higher levels of plasma cells, CD8+ T cells, regulatory T cells (Tregs), activated natural killer (NK) cells, and dendritic cells (DCs) compared to cluster A. For instance, tumor cells stimulate lactic acid production through LDHA, which disrupts the secretion of IFN-γ and other cytokines in tumor-infiltrating T cells and NK cells, thereby promoting tumor growth (55). Alternatively, it modulates RNA methyltransferase METTL3-mediated myeloid cell infiltration into tumors via protein lactation. N6-methyladenosine (m6A) modification of TIMs enhances their immunosuppressive function (54). However, within the TME, the impact of lactate on both tumors and immune cells can be confounded by acidic protons generated during glycolysis. To eliminate interference from protons dissociated from its acidic counterpart - lactic acid - sodium lactate was investigated for its effect on immunity. This study suggests that the influence of lactic acid on immunity is two-fold; lactate can enhance CD8+ T cell dryness (functionality), thus augmenting anti-tumor immune response (56).

Additionally, there were notable variations observed in the abundance of immune cell infiltration between geneCluster A and geneCluster B. It is worth mentioning that seven different cell types exhibited statistically significant variances (*p<0.05*). For instance, geneCluster A demonstrated a higher prevalence of CD8+T cells and activated NK cells, which were associated with a more favorable prognosis. Conversely, geneCluster B showed an increased presence of M2 macrophages. Additionally, a study elucidated the interplay between pituitary adenoma (PA) cells and the immune tumor microenvironment (TME), highlighting their role in promoting PA invasion through M2 polarization. Furthermore, lactate levels demonstrated a positive correlation with PA invasion (57).

In recent years, immunotherapy has effectively changed the treatment landscape of OV (58). Immune checkpoint inhibitors (ICIs) have shown clinical benefit for patients with OV. However, OV patient’s response to immunotherapy is limited (59). Thus, the evaluation of sensitive/resistant target treatment subpopulations based on stratification by tumor biomarkers may improve the predictiveness of response to immunotherapy. By analyzing the immunotherapy datasets, we proved that patients with high lactate score were resistant to ICI treatment in GSE126044 cohorts.

In summary, we initially defined genes that were differentially expressed in lactate-related pathways as LMRGs and subsequently stratified OV patients into two distinct clusters, exhibiting significant disparities in terms of survival prognosis, and immune infiltration, among other factors. Furthermore, we additionally developed the lactate score as an independent prognostic indicator for predicting the outcome of OV patients. These findings highlight the important clinical significance of LMRGs and provide a new idea for guiding the personalized treatment strategy of OV patients.

## Data availability statement

The datasets presented in this study can be found in online repositories. The names of the repository/repositories and accession number(s) can be found below: TCGA OV RNAseq dataset (https://portal.gdc.cancer.gov/), GEO (https://www.ncbi.nlm.nih.gov/geo/query/acc.cgi?acc=GSE140082), EBI (https://www.ebi.ac.uk/), MsigDB (https://www.gsea-msigdb.org/gsea/msigdb).

## Author contributions

XCheng: Conceptualization, Funding acquisition, Resources, Supervision, Writing – original draft, Writing – review & editing. LZ: Data curation, Methodology, Software, Writing – original draft. ZL: Formal analysis, Investigation, Project administration, Validation, Writing – original draft. KW: Formal analysis, Methodology, Software, Writing – original draft. JG: Data curation, Methodology, Writing – original draft. XChen: Methodology, Writing – original draft. RC: Formal analysis, Methodology, Writing – original draft. LW: Conceptualization, Writing – review & editing.
